# Spontaneous Retroperitoneal Hemorrhage Treated With Transcatheter Arterial Embolization in a Patient With Acquired Cystic Kidney Disease: A Case Report

**DOI:** 10.7759/cureus.78532

**Published:** 2025-02-05

**Authors:** Halleluyah Konno, Masakazu Nitta, Yoichi Iwafuchi, Yuko Oyama, Norihiro Watanabe

**Affiliations:** 1 Emergency Medicine, Kenoh Kikan Hospital, Sanjo, JPN; 2 Internal Medicine, Kenoh Kikan Hospital, Sanjo, JPN

**Keywords:** acquired cystic kidney disease (ackd), maintenance hemodialysis, spontaneous renal rupture, spontaneous retroperitoneal hemorrhage, transarterial embolization (tae)

## Abstract

Spontaneous retroperitoneal hemorrhage is a rare condition with a poor prognosis. It is usually treated conservatively unless vital signs are unstable. We report a case of a 60-year-old male patient with acquired cystic kidney disease who presented to ED due to continuous left lower abdominal pain. His vital signs were normal, and a physical examination revealed tenderness in the lower left abdomen. CT revealed a hematoma with extravasation around the left kidney. He was treated conservatively due to stable vital signs and laboratory data. However, a decrease in the hemoglobin level and an increase in hematoma were observed, and blood transfusion and transcatheter arterial embolization were performed on the second day of admission. In such cases, early embolization should be considered even when the patient's general condition is stable.

## Introduction

Spontaneous retroperitoneal hemorrhage (SRH) is a rare condition. Acquired cystic kidney disease (ACKD), which is often observed in patients on long-term hemodialysis, has been reported to be a more likely cause of SRH due to tissue fragility and hemorrhagic tendency [1]. Unless the vital signs are unstable, SRH is treated conservatively for two reasons: first, the hematoma being packed in the retroperitoneum even if extravasation is seen on CT, and second, due to the functional prognosis of the kidney itself.

Herein, we describe a case of SRH in a patient with ACKD who initially received conservative treatment but later required blood transfusion and transcatheter arterial embolization (TAE).

## Case presentation

A 63-year-old man was presented to the ED after undergoing hemodialysis. He was diagnosed with chronic renal failure due to mitochondrial nephropathy at the age of 38 years and has visited the hospital three times a week for dialysis since then. On the morning of his visit, he experienced left abdominal pain during hemodialysis. He decided to wait and see because the pain was under control. He decided to visit the ER after dialysis because of increased pain.

**Figure 1:**
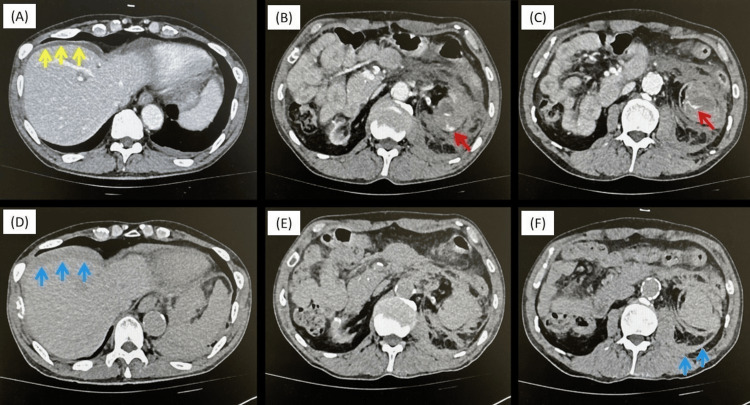
CT of the abdomen A-C, CT at the ER department. Extravasation from the lower branch of the renal artery (red arrow) and hematoma around the liver (yellow arrow) are observed. D-F, same slices with A-C of CT taken on the second day of hospitalization. An increase of hematoma around the liver and left lower pole of the kidney was observed (blue arrow).

His vital signs on arrival were as follows: heart rate, 75 bpm; blood pressure, 128/71 mmHg; SpO_2_ stable at 100% without oxygenation; and Glasgow Coma Scale, 15. Physical examination revealed tenderness in the left lower abdomen; however, no rebound tenderness or guarding was observed. Blood tests showed no progression of anemia or significant changes in the coagulation system compared with those before dialysis (Table 1). A contrast-enhanced abdominal CT revealed a hematoma with extravasation around the left kidney, leading to a diagnosis of left renal hematoma secondary to possible cyst rupture (Figures 1A-1C). Since the vital signs were stable, the patient was admitted for observation, but urgent hemostasis was not performed. Vital signs remained stable on the second day of hospitalization. However, blood tests showed a decreased hemoglobin (Hb) level of 6.2, and a transfusion of four units of concentrated RBCs was administered (Table 1). CT showed residual extravasation at the left lower pole of the kidney and an increased hematoma around the liver (Figures 1D-1F). Considering the patient's history of antithrombotic medication use and exacerbation of bleeding due to anticoagulant use during dialysis, TAE was performed.

**Table 1:** Laboratory findings of the patient before hemodialysis, visit at the ER department, and on the second day of hospitalization APTT: Activated partial thromboplastin time; BUN: Blood urea nitrogen; Ca: Calcium; Cl: Chloride; Cre: Creatinine; CRP: C-reactive protein; ER: Emergency room; Hb: Hemoglobin; Hct: Hematocrit; K: Potassium; Na: Sodium; Plt: Platelets; PT–INR: Prothrombin time–international normalized ratio; RBC: Red blood cell; WBC: White blood cell.

Laboratory data	Normal values	Before hemodialysis	ER department	Second day at the hospital
					BUN (mg/dL)	8.0-22.0	26.9	25.6	31.0
Cre (mg/dL)	0.6-1.1	5.46	6.01	7.02
Na (mmol/L)	138-146	138	139	138
K (mmol/L)	3.6-4.9	3.1	4.2	4.0
Cl (mmol/L)	99-109	105	102	102
Ca (mg/dL)	8.7-10.3	7.9	8.3	7.7
CRP (mg/dL)	<0.30	0.08	0.24	N/A
WBC (/uL)	4000-8800	9930	7810	7180
RBC (x 10^4^/uL)	420-550	260	245	196
Hb (g/dL)	13.5-18.0	8.3	8.2	6.3
HCT (%)	36.0-54.0	26.2	25.2	19.9
PLT (x 10^4^/uL)	15.0-35.0	12.2	11.0	8.5
PT–INR	0.90-1.14	N/A	0.80	0.92
APTT (sec)	24-40	N/A	25.9	27.7

**Figure 2:**
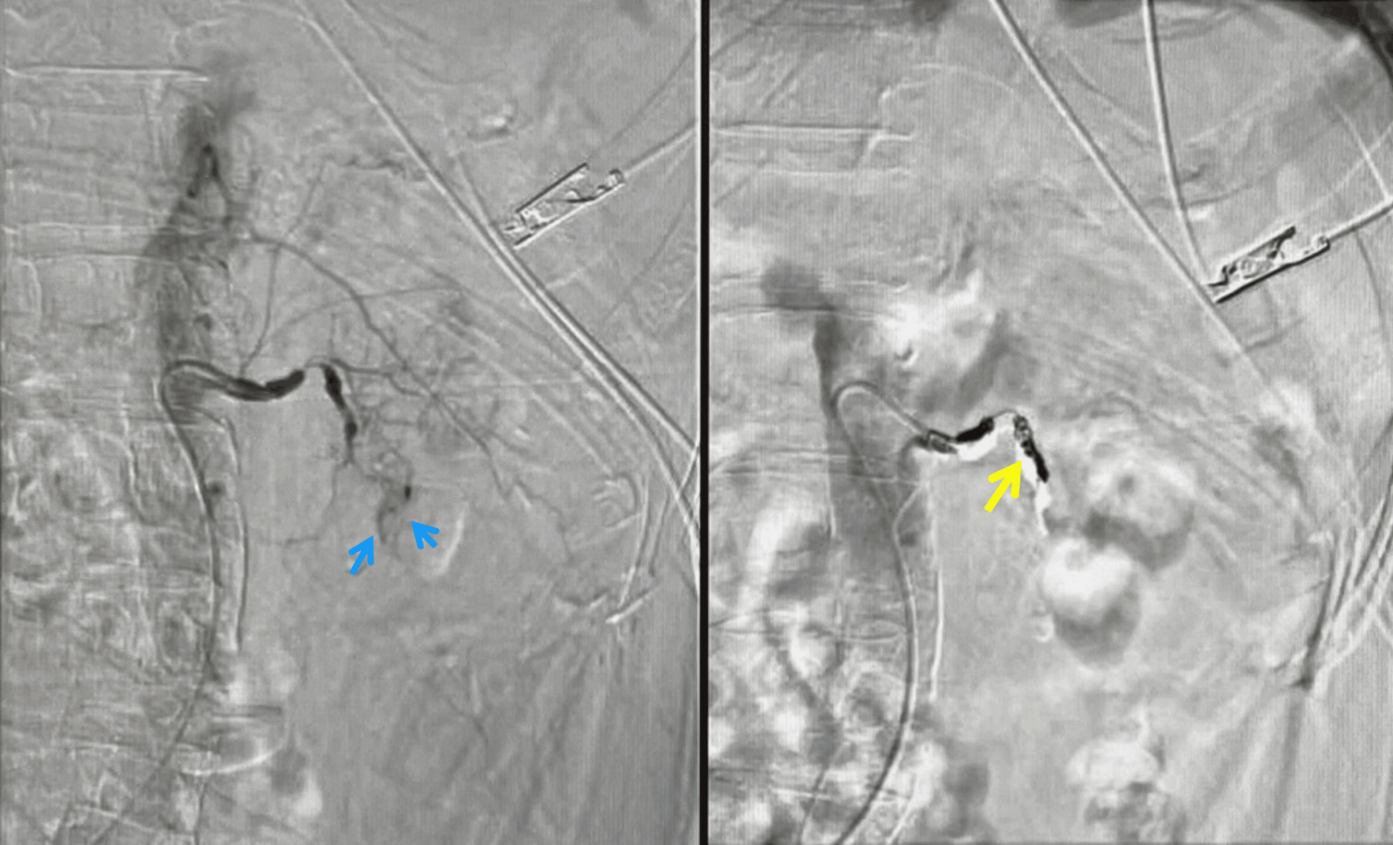
TAE findings Extravasation is observed at the lower pole of the kidney (blue arrow). Embolization was performed using a sponsel and coil (yellow arrow).

The left renal artery was narrowed at the distal portion of the main trunk, and a small amount of contrast medium leakage was observed at the left lower pole of the kidney (Figure 2, blue arrow). A microcatheter was inserted into the branch of the left inferior pole of the renal artery, and after inflow of the sponsel strip, embolization was performed using a 3-4 mm diameter coil. In addition, a small amount of sponsel was inserted from the main trunk of the left renal artery, and the main trunk was embolized using a 5-6 mm diameter coil (Figure 2, yellow arrow).

Hemodialysis was resumed on the third day of hospitalization. Considering the patient's post-hemorrhagic state, nafamostat (Fusan) was administered instead of heparin. The pre-dialysis blood test showed Hb 7.4, and an additional transfusion of two units of concentrated RBC was administered. From the fourth day of hospitalization onward, no progression of anemia was observed. On the 28th day of hospitalization, CT showed no extravasation, and the perinephric hematoma was absorbed. He was discharged on the 29th day of hospitalization. Since then, he visits our hospital for hemodialysis, without any recurrence of abdominal pain.

## Discussion

Recent advances in diagnostic imaging have revealed a high incidence of multiple cysts in the kidneys of long-term hemodialysis patients, referred to as ACKD. Ishikawa et al. reported that a high rate of 47.1% of hemodialysis patients in Japan have ACKD [1], and Degrassi F et al. reported that the incidence of ACKD is proportional to the duration of dialysis, with 90% of patients on hemodialysis for more than 10 years developing ACKD [2]. Although SRH is rare, Otsuki et al. reported that 7 of 66 patients (10.6%) died from SRH, and appropriate early treatment for SRH is necessary [3]. It is reported that the frequency of SRH in long-term dialysis patients is about 3% [4], and ACKD may be a risk factor for SRH [5]. Therefore, SRH should be included in the differential diagnosis of sudden onset abdominal or back pain in long-term dialysis patients.

The first-line treatment for SRH remains controversial because the kidneys are covered by Gerota's fascia. Even if bleeding progresses to some degree, it will be compressed and hemostatic within the fascia. Therefore, conservative treatments such as analgesia and blood transfusion are possible when no abnormal vital signs or progressive anemia are observed. However, TAE or surgical treatment should be considered if hypotension or progressive anemia is observed. Around 30% of patients with SRH have concurrent malignant tumors [6], and since multiple sites of hemorrhage are not uncommon, some reports recommend nephrectomy for radical treatment [7,8]. However, surgical treatment is associated with a high mortality rate. In fact, Bensalah et al. recommended nephrectomy but reported a high mortality rate of 38% in patients with idiopathic renal rupture with ACKD who underwent nephrectomy [9]. Chen et al. reported that 17 of 18 patients with ACKD and spontaneous renal rupture who underwent TAE had successful hemostasis with TAE alone and did not require rebleeding or surgical intervention [10]. This suggests that TAE is effective even in atrophic kidneys that do not require functional preservation.

In our case, because there was no obvious evidence of a renal tumor on CT and there was no decrease in blood pressure or Hb level, conservative treatment was chosen, although extravasation was observed. However, anemia developed, and a follow-up CT scan showed increased hematoma size; therefore, RBC transfusion and TAE were performed the next day. If it is difficult to respond to the situation, asking for a urologist or a transfer to a hospital that could handle the situation could be a solution. However, owing to the tissue fragility of ACKD, the possibility of prolonged active bleeding, and the need to use anticoagulants for continuous hemodialysis, swift intervention is needed, and TAE could be a better solution when the patient presents to the ED.

## Conclusions

We encountered a case of SRH in a patient with ACKD treated with TAE. SRH needs to be treated swiftly; however, the first-line treatment for SRH remains controversial. In such cases, early embolization should be considered even when the patient's general condition is stable.
